# The Effect of Hospital Volume on Mortality in Patients Admitted with Severe Sepsis

**DOI:** 10.1371/journal.pone.0108754

**Published:** 2014-09-29

**Authors:** Sajid Shahul, Michele R. Hacker, Victor Novack, Ariel Mueller, Shahzad Shaefi, Bilal Mahmood, Syed Haider Ali, Daniel Talmor

**Affiliations:** 1 Department of Anesthesiology, Beth Israel Deaconess Medical Center, Harvard Medical School, Boston, Massachusetts, United States of America; 2 Department of Obstetrics and Gynecology, Beth Israel Deaconess Medical Center, Harvard Medical School, Boston, Massachusetts, United States of America; 3 Clinical Research Centre, Soroka University Medical Centre and Faculty of Health Sciences, Ben-Gurion University of the Negev, Beer-Sheva, Israel; 4 Washington and Lee University, Lexington, Virginia, United States of America; Hospital Sirio-Libanes, Brazil

## Abstract

**Importance:**

The association between hospital volume and inpatient mortality for severe sepsis is unclear.

**Objective:**

To assess the effect of severe sepsis case volume and inpatient mortality.

**Design Setting and Participants:**

Retrospective cohort study from 646,988 patient discharges with severe sepsis from 3,487 hospitals in the Nationwide Inpatient Sample from 2002 to 2011.

**Exposures:**

The exposure of interest was the mean yearly sepsis case volume per hospital divided into tertiles.

**Main Outcomes and Measures:**

Inpatient mortality.

**Results:**

Compared with the highest tertile of severe sepsis volume (>60 cases per year), the odds ratio for inpatient mortality among persons admitted to hospitals in the lowest tertile (≤10 severe sepsis cases per year) was 1.188 (95% CI: 1.074–1.315), while the odds ratio was 1.090 (95% CI: 1.031–1.152) for patients admitted to hospitals in the middle tertile. Similarly, improved survival was seen across the tertiles with an adjusted inpatient mortality incidence of 35.81 (95% CI: 33.64–38.03) for hospitals with the lowest volume of severe sepsis cases and a drop to 32.07 (95% CI: 31.51–32.64) for hospitals with the highest volume.

**Conclusions and Relevance:**

We demonstrate an association between a higher severe sepsis case volume and decreased mortality. The need for a systems-based approach for improved outcomes may require a high volume of severely septic patients.

## Background

The mortality from severe sepsis remains unacceptably high despite available treatment. It is estimated that 34% of all patients hospitalized with sepsis have severe sepsis and that inpatient mortality approaches 40% among this patient population [1]. Treatment for severe sepsis requires an aggressive, sophisticated, complex and multidisciplinary approach to improve patient outcomes. Diagnostic and therapeutic tasks need to be performed in a specific time frame to achieve optimal survival. These actions are outlined in the Surviving Sepsis Campaign guidelines [2]. Adherence to these guidelines has led to a decrease in hospital mortality [3], [4]. The need for a systems-based approach with aggressive adherence may require a high volume of severely septic patients to ensure successful implementation with a low failure rate.

There is a well-documented relationship between hospital volume and patient outcome. Higher patient volumes are associated with improved mortality in complex, high-risk oncologic, cardiac and bariatric surgical procedures [5], [6], [7]. This relationship between hospital volume and mortality is also seen with common medical conditions as well, for example acute myocardial infarction, heart failure and pneumonia [8]. The strong demonstration of volume-outcome relationships in both relatively common medical conditions and high-risk surgical conditions may be reflective of increasing provider sophistication, leading to improved quality indicators such as shorter length of stay and decreased risk-adjusted complications.

Previous studies examining the association between hospital volume and patient mortality for severe sepsis have had conflicting results. Shahin, et al demonstrated no difference in mortality between patients admitted to low-volume or high-volume critical care units in the United Kingdom [9]. Reinikanien and colleagues however did demonstrate that patients admitted with severe sepsis to lower-volume Finnish critical care units had higher mortality than those admitted to larger units [10]. It has been suggested that the Shahin study may have been underpowered to detect differences whereas the Reinikanien study may have been biased due to the lack of adjustment for clustering [10]. More recently Walkey et al demonstrated volume-outcome differences in patients admitted with severe sepsis to academic medical centers in the United States. The generalizability of this study may be a concern given the exclusion of rural and non-academic community hospitals [11].

Hence, the relationship between severe sepsis hospital volume and mortality remains unclear. Mortality reductions have been observed in admissions to higher-volume hospitals for both medical and surgical conditions that influence outcomes in severe sepsis [12]. In addition, factors that influence outcome in severe sepsis, such as mechanical ventilation, heart failure and pneumonia, are all influenced by hospital volume [8], [12].

We hypothesized that there is a relationship between hospital characteristics and mortality associated with severe sepsis nationwide. We used a national administrative database to examine whether hospital characteristics influenced inpatient mortality in patients with severe sepsis.

## Methods

### Data Source

We performed a retrospective cohort study using a nationally representative administrative database - the Nationwide Inpatient Sample (NIS) from 2002 to 2011. The NIS of the Healthcare Cost and Utilization Project is the largest administrative database of inpatient care in the United States and provides data on approximately 8 million hospital stays annually. It contains discharge data from 1000 short-term and non–federal hospitals, which represent a 20%, stratified sample of patient-level data in participating hospitals. These hospitals include teaching, non-teaching and rural hospitals. The sampling frame of NIS comprises approximately 97% of all hospital discharges in the United States. The NIS dataset includes patient demographics and comorbidities, hospital characteristics, inpatient mortality and disposition. Since the NIS has no patient identifiers, the Committee on Clinical Investigations at Beth Israel Deaconess Medical Center declared this study exempt.

Validations of data elements are performed annually in the NIS database with both internal and external quality assessments. External validation has performed well when the NIS dataset has been compared against the American Hospital Association Annual Survey database, the National Hospital Discharge Survey from the National Center for Health Statistics and the MedPAR inpatient data from the Centers of Medicare and Medicaid Services.

### Study Population

The study population included all patients with a discharge diagnosis of severe sepsis from 2002 through 2011. We identified patients with severe sepsis by the presence of the *International Classification of Diseases*, Ninth Revision (ICD-9-CM) code 995.92 in the discharge diagnosis. This ICD-9-CM code is the only code for severe sepsis that has previously been validated in the NIS against institutional data. Validation studies based on this case definition have demonstrated moderate sensitivity (52%, 95% CI: 39–65%), a high specificity (98%, 95% CI: 92–100%) and very high positive predictive value (approximately 100%) [13], [14]. To address that transfer to a high volume center may represent a competing outcome we excluded patients transferred to any acute care facility. To address that large volume centers may transfer early to skilled nursing facilities, which may bias outcomes, we performed a sensitivity analysis with and without transfer to skilled nursing facilities.

### Sensitivity Analyses

To test the robustness of our results we used alternative ICD- 9 coding algorithms for severe sepsis developed by Angus. This algorithm has reported sensitivity of 50.3% with a high specificity of 96.3%. This algorithm has been validated against patient level data similar to the severe sepsis algorithm that we used above [14]. We conducted a volume outcome relationship among patients admitted with severe sepsis.

### Exposure, Outcome and Covariates

The exposure of interest was the mean yearly sepsis case volume per hospital divided into tertiles. We chose tertiles, to ensure a meaningful range of sepsis cases within each group, particularly in the lowest volume category. The primary outcome was inpatient mortality. Inpatient mortality included mortality in the hospital, skilled nursing facility, intermediate care facility or freestanding hospice. Characteristics that were considered as potential confounders to be included in the model as covariates included age, race, sex, teaching status, hospital region, median household income for the patient's zip code. To adjust for comorbid conditions, we used the Charlson Comorbidity Index, calculated by weighting comorbidities. Previous work has demonstrated that the Charlson Comorbidity Index has excellent discriminative power to predict inpatient mortality in administrative databases [15].

### Statistical Analyses

All analyses were performed using SAS 9.3 (SAS Institute, Cary, NA) and SUDAAN 10.0 (Research Triangle Institute, Research Triangle Park, NC) to account for the complex survey design of the NIS. Frequencies and proportions were calculated and weighted to reflect national estimates. NIS data can be weighted to produce national level estimates by utilizing standard stratum-specific discharge weights provided by the Healthcare Cost and Utilization Project. Weighted estimates were used for the analyses to produce accurate unbiased estimates. Categorical variables are presented as frequencies and proportions and were compared using the Chi-square test. We compared inpatient mortality by fitting multivariate logistic regression models sequentially, using generalized estimating equations with robust variance estimates to account for within-hospital clustering, to calculate odds ratios (OR) and 95% confidence intervals (CI). We first fit an unadjusted logistic regression model to estimate the OR for inpatient mortality among patients with severe sepsis relative to tertiles of annual hospital volume for severe sepsis. We then fit a multivariate adjusted model, adjusting for patient age, sex, race, Charslon Comorbidity Index, median household income for the patient's zip code, teaching status and hospital location. All tests were two sided and p-values <0.05 were considered statistically significant.

## Results

### Demographic and Clinical Characteristics

We identified 646,988 patient discharges with severe sepsis from 2002 to 2011, corresponding to a weighted estimate of 3,179,092 discharges. Women accounted for 49.24% of patients with severe sepsis, and the majority of patients were white (68.73%) with 15.32% black, 9.88% Hispanic, 3.05% Asian, and 0.61% Native American. There were 3,487 hospitals in the NIS dataset, and the median annual volume of severe sepsis cases was 50. The tertiles for annual volume of severe sepsis were 10 or fewer cases per year, 11 to 60 cases per year, and greater than 60 cases per year; the three tertiles included 1,170, 1,155 and 1,162 hospitals, respectively.

Patients in the highest tertile of severe sepsis volume were more likely to be younger, non-white and female. They also had a higher severity of illness as indicated by Charlson scores, as compared to patients admitted in the other two tertiles. The highest volume tertile had a higher proportion of patients with valvular disease, pulmonary circulation disease, renal failure diabetes, liver disease, obesity and neurological disease. Patient characteristics stratified by tertile of severe sepsis volume are shown in Table 1.

**Table 1:** Patient Characteristics at Presentation.

		Annual Hospital Volume of Severe Sepsis *N (Weighted N) %*			P
		≤10 Cases	11–60 Cases	>60 Cases	
**No. of Patients (%)**		8941 (1.38)	85754 (13.25)	552293 (85.36)	
***Characteristics***					
**Age (yrs)**	**<18**	164 (763) 1.77	1667 (8082) 2.00	7772 (38465) 1.48	<0.0001
	**18–44**	502 (2489) 5.79	5727 (27889) 6.92	46635 (228824) 8.81	
	**45–64**	1906 (9477) 22.04	21502 (105267) 26.11	153844 (755778) 29.10	
	**65–84**	4082 (20542) 47.77	38326 (187969) 46.63	233895 (1149367) 44.26	
	**85–100**	1906 (9500) 22.09	14518 (71351) 17.70	83099 (408809) 15.74	
	**≥100**	47 (229) 0.53	534 (2570) 0.64	3187 (15844) 0.61	
**Race**	**White**	5391 (27077) 80.73	53003 (260248) 75.97	319682 (1571465) 67.08	<0.0001
	**Black**	554 (2798) 8.34	7464 (36552) 10.67	75623 (371887) 15.88	
	**Hispanic**	306 (1544) 4.60	5390 (26061) 7.61	49172 (240932) 10.29	
	**Asian/Pacific Islander**	95 (479) 1.43	1206 (5921) 1.73	15829 (76427) 3.26	
	**Native American**	106 (516) 1.54	955 (4739) 1.38	2392 (11932) 0.51	
	**Other**	216 (1125) 3.35	1825 (9053) 2.64	14073 (69866) 2.98	
**Sex**	**Female**	4243 (21240) 47.61	42241 (207241) 49.33	281433 (1383711) 50.98	<0.0001
	**Male**	4688 (23369) 52.39	43509 (212898) 50.67	270835 (1330437) 49.02	
***Comorbidities***					
** Congestive Heart Failure**		2152 (10735) 24.04	21282 (104054) 24.77	133078 (654318) 24.11	0.25
** Valvular Disease**		334 (1672) 3.75	4019 (19672) 4.68	26947 (132480) 4.88	0.0001
** Peripheral Vascular Disease**		384 (1944) 4.35	4505 (22142) 5.27	33227 (163471) 6.02	<0.0001
** Other Neurological Disorders**		1079 (5372) 12.03	9438 (466166) 10.99	61647 (303182) 11.17	0.10
** Chronic Pulmonary Disease**		1886 (9408) 21.07	20581 (100584) 23.94	118288 (581731) 21.43	<0.0001
** Chronic Diabetes with Complications**		391 (1974) 4.42	4373 (21387) 5.09	31793 (156184) 5.75	<0.0001
** Renal Failure**		1553 (7894) 17.68	17183 (84246) 20.05	125229 (615153) 22.66	<0.0001
** Liver Disease**		265 (1330) 2.98	3333 (16246) 3.87	28318 (139055) 5.12	<0.0001
** Obesity**		341 (1713) 3.84	3697 (18165) 4.32	27715 (135678) 5.00	<0.0001
**Charlson Comorbidity Index**	**0**	2777 (13905) 31.14	23231 (113990) 27.13	127983 (628523) 23.16	<0.0001
	**1**	2729 (13543) 30.33	24129 (118104) 28.11	142901 (702384) 25.88	
	**2**	1892 (9485) 21.24	18399 (89995) 21.42	121904 (599387) 22.08	
	**3**	775 (3880) 8.69	9305 (45576) 10.85	67937 (333857) 12.30	
	**4**	285 (1422) 3.18	3991 (19632) 4.67	32726 (160730) 5.92	
	**≥5**	483 (2422) 5.42	6699 (32859) 7.82	58842 (289396) 10.66	

Hospitals in the lowest tertile were more likely to be small, rural hospitals with patients who had a median household income at or below the 25^th^ percentile. There also was significant geographic variation, with a small proportion of these hospitals being located in the northeast or west compared with hospitals in the two higher volume tertiles. Hospital characteristics stratified by tertile of severe sepsis volume are shown in Table 2.

**Table 2:** Hospital characteristics.

		Annual Hospital Volume of Severe Sepsis *N (Weighted N) %*			P
		≤10 Cases	11–60 Cases	>60 Cases	
**No. of Patients (%)**		8941 (1.38)	85754 (13.25)	552293 (85.36)	
**Characteristics**					
**Bed Size of Hospital**	**Small**	5188 (25900) 58.47	26833 (128420) 30.75	36908 (166896) 6.21	<0.0001
	**Medium**	2265 (11023) 24.89	30135 (147667) 35.36	124531 (616657) 22.93	
	**Large**	1411 (7373) 16.64	28259 (141530) 33.89	385720 (1905798) 70.86	
**Transfer in from Another Acute Care Hospital**		506 (2624) 11.36	3848 (19505) 8.41	32089 (156620) 8.59	0.0006
**Teaching Status**	**Rural**	5122 (26116) 58.96	24421 (121550) 29.11	20743 (100829) 3.75	<0.0001
	**Urban Non-Teaching**	3315 (16136) 36.43	47787 (233313) 55.87	231530 (1129292) 41.99	
	**Urban Teaching**	427 (2044) 4.61	13019 (62753) 15.03	294886 (1459230) 54.26	
**Hospital Location**	**Rural**	5122 (24966) 57.59	24421 (118656) 28.54	20743 (100845) 3.79	<0.0001
	**Urban**	3742 (18388) 42.41	60806 (297083) 71.46	526416 (2561156) 96.21	
**Hospital Region**	**Northeast**	877 (4255) 9.73	15825 (76863) 18.37	110516 (537772) 20.01	<0.0001
	**Midwest**	3037 (14964) 34.22	19273 (94606) 22.62	107196 (521730) 19.42	
	**South**	3844 (18837) 43.07	36079 (176711) 42.24	198164 (967411) 36.00	
	**West**	1183 (5675) 12.98	14577 (70128) 16.76	136417 (660155) 24.57	
**Median Household Income for Patient's Zip code (percentile)**	**0–25th**	3758 (18897) 44.27	27370 (134625) 33.08	148141 (728981) 27.53	<0.0001
	**26th–50th**	2916 (14512) 34.00	24452 (119369) 29.33	130077 (639772) 24.16	
	**51st–75th**	1431 (7052) 16.52	18821 (91991) 22.60	133765 (656282) 24.78	
	**76th–100th**	444 (2226) 5.22	12411 (61034) 15.00	126969 (623011) 23.53	

### Inpatient Mortality

The crude overall inpatient mortality rate was 33.36%. After adjusting for age, race, sex, Charslon Comorbidity score, teaching status, hospital region, and median household income for the patient's zip code lower hospital volume was associated with a significant increase in the risk of inpatient mortality. Compared with the highest tertile of severe sepsis volume (>60 cases per year), the odds ratio for inpatient mortality among persons admitted to hospitals in the lowest tertile (≤10 severe sepsis cases per year) was 1.188 (95% CI: 1.074–1.315), while the odds ratio was 1.090 (95% CI: 1.031–1.152) for patients admitted to hospitals in the middle tertile (Table 3). Similarly, improved survival was seen across the tertiles with an adjusted inpatient mortality incidence of 35.81 (95% CI: 33.64–38.03) for hospitals with the lowest volume of severe sepsis cases and a drop to 32.07 (95% CI: 31.51–32.64) for hospitals with the highest volume (Table 3). In the multivariate analysis for mortality, we found a significant increase in mortality with increasing age and charlson score. (Table 4). In our cohort of patients with a diagnosis of severe sepsis, 53.01% had an associated secondary diagnosis of septic shock. The proportion with septic shock was 53.25% in the highest tertile, followed by 51.78% in the middle tertile and 50.05% in the lowest tertile. The crude overall mortality in this subgroup of patients was 39.80%. The crude overall mortality for patients diagnosed with severe sepsis but without shock was 27.54%.

**Table 3:** Association between Hospital Volume and Risk-Adjusted Mortality.

	Annual Hospital Volume of Severe Sepsis		
	≤10 Cases	11–60 Cases	>60 Cases
**No. of Hospitals (%)**	1170 (33.55)	1155 (33.12)	1162 (33.32)
**Odds Ratio (95% CI)**			
Unadjusted Model	1.066 (1.003, 1.133)	1.059 (1.020, 1.100)	1.0 [Reference]
Demographics Adjusted Model^1^	1.131 (1.024, 1.250)	1.077 (1.018, 1.138)	1.0 [Reference]
Multivariate Model Adjusting for Severity^2^	1.188 (1.074, 1.315)	1.090 (1.031, 1.152)	1.0 [Reference]
**Mortality Incidence (95% CI)**			
Unadjusted Model	35.53 (34.23, 36.84)	35.37 (34.68, 36.07)	34.07 (33.59, 34.55)
Demographics and Comorbidity Adjusted Model^1^	34.29 (32.17, 36.47)	33.21 (32.12, 34.32)	31.62 (31.06, 32.18)
Multivariate Model Adjusting for Severity^2^	35.81 (33.64, 38.03)	33.91 (32.82, 35.02)	32.07 (31.51, 32.64)

*CI: Confidence Interval.*

1 *Adjusted for age group, race, median income, sex, teaching status, and hospital region.*

2 *Adjusted for Charlson score, age group, race, median income, sex, teaching status, and hospital region.*

**Table 4:** Multivariate Model Adjusting for demographics and severity of illness.

Variables	Odds Ratio (95% CI)
**Sepsis Tertiles**	
>60 Cases	1.0 [Reference]
10–60 Cases	1.090 (1.031, 1.152)
≤10 Cases	1.188 (1.074, 1.315)
**Charlson Score**	
0	1.0 [Reference]
1	1.061 (1.037, 1.085)
2	1.160 (1.128, 1.192)
3	1.230 (1.193, 1.268)
4	1.544 (1.484, 1.606)
≥5	2.550 (2.467, 2.636)
**Median Household Income for Patient's Zip Code**	
0–25th percentile	1.0 [Reference]
26th to 50th percentile (median)	1.003 (0.978, 1.028)
51st to 75th percentile	1.006 (0.980, 1.033)
76th to 100th percentile	1.045 (1.016, 1.075)
**Age Group**	
<18	1.0 [Reference]
18–44	1.043 (0.946, 1.149)
45–64	1.451 (1.318, 1.598)
65–84	2.184 (1.979, 2.411)
85–100	3.985 (3.605, 4.404)
≥100	7.414 (5.745, 9.566)
**Race**	
White	1.0 [Reference]
Black	0.952 (0.928, 0.976)
Hispanic	0.977 (0.945, 1.009)
Asian/Pacific Islander	0.992 (0.950, 1.037)
Native American	0.922 (0.839, 1.014)
Other	0.979 (0.929, 1.032)
**Female sex**	1.051 (1.035, 1.067)
**Teaching Status**	
Rural	1.0 [Reference]
Urban Non-Teaching	0.910 (0.851, 0.973)
Urban Teaching	1.033 (0.958, 1.114)
**Hospital Region**	
Northeast	1.0 [Reference]
Midwest	0.763 (0.712, 0.817)
South	0.977 (0.923, 1.034)
West	0.861 (0.808, 0.918)

### Sensitivity Analyses

An analysis excluding all patients discharged to skilled nursing facility or intermediate care facility or short-term hospital showed similar associations between mean severe sepsis hospital volume and mortality. The odds ratio comparison between the highest and lowest tertiles was 1.56 (95% CI: 1.43–1.71), while the odds ratio for the middle tertile was 1.23 (95% CI: 1.18–1.29).

Using the Angus algorithm for the identification of severe sepsis patients, the odds ratio comparison was similar to the primary analysis. The odds ratio comparison between the highest and lowest quartiles was 1.21 (95% CI: 1.12–1.32), while the odds ratio for the middle tertile was 1.17 (95% CI: 1.11–1.22).

## Discussion

The principal findings of this study demonstrate a significant association between increasing hospital volume of severe sepsis cases and reduced risk of inpatient mortality. This effect was visible both in the crude analysis and after adjustment for relevant confounders.

There are multiple reasons for an association between hospital volume and mortality in patients with severe sepsis. This likely is a result of increased provider expertise coupled with improved adherence to best practice guidelines. Previous studies have noted that participation in multi-dimensional evidenced-based initiatives, such as use of the Surviving Sepsis Campaign guidelines, has led to a mortality reduction of 5.4% over 2 years [2].

Intensive care unit organizational structure and staffing could also account for the differences in our observed mortality. In our analysis, hospitals in the highest severe sepsis volume tertile were likely to be urban teaching or community hospitals. Recent data suggests that patients in these hospitals were likely to be cared for by a dedicated housestaff team, working with an intensivist in a closed intensive care unit [16]. Furthermore, these hospitals have rapid response teams that attend to and triage rapidly decompensating patients. These hospitals are also more likely to have daily structured care plans and protocols for ventilator management in contrast to the lowest tertile hospitals [17].

Recent work by Walkey et al demonstrated similar volume-outcome differences in patients with severe sepsis admitted to US academic hospitals. However, despite the similarity in outcome between that study and ours, there are important differences that need to be highlighted [11]. We chose to use the NIS, which represents approximately 20% of all hospital discharges in the US. These hospitals are characterized as rural, urban non–teaching and urban teaching. Most teaching and urban community hospitals in the Walkey et al study (96%) would fall in the highest severe sepsis volume tertile in our analysis, thereby not accounting for most rural hospitals (86%) that fall in the two lowest volume tertiles. This is likely the reason we demonstrate higher odds ratios in the comparison between tertiles, as our study includes both rural and urban hospitals (teaching and non-teaching).

Our work differs from previous work by Kumar et al who examined trends in severe sepsis using the NIS. They found no differences in hospital mortality when comparing small, medium and large hospitals. This may be due to the fact that the definition of hospital size varies with the geographic region in the NIS, thus making hospital size a poor surrogate for hospital volume [18].

Optimal survival in severe sepsis is predicated upon early, appropriate antimicrobial therapy, aggressive hemodynamic resuscitation and laboratory vigilance to achieve resuscitation goals. Previous studies demonstrated a similar volume-outcome relationship among sepsis admissions in hospital emergency departments. Powell and colleagues demonstrated that there was a 31% decrease in mortality between the highest quartile versus the lowest quartile emergency departments (ED) [19]. Given the fact that most high volume EDs were in teaching hospitals, the authors hypothesized that the difference in mortality was likely due to better resuscitation at larger EDs. The converse also has been demonstrated in that rural EDs rarely meet complete Surviving Sepsis Campaign guidelines due to the absence of technical and administrative expertise [20]. Recognition of inadequate therapy due to a lack of technical expertise and appropriate infrastructure has prompted some hospital systems to create tiered sepsis checklists that target basic stabilization before transfer [20].

The rationale for the ICD-9-CM code that we used for the identification of severe sepsis, 995.92, was the validation against institutional data with moderate sensitivity and very high specificity. Validation for severe sepsis specifically from the NIS dataset has demonstrated a sensitivity of 52% and a specificity of 98% [13]. Though the reported sensitivity is moderate, the large sample size with a very high specificity makes this an appropriate sepsis cohort for the study. The sensitivity analysis based on the Angus algorithm demonstrated similar results to our primary analysis thus making our results more robust.

The mortality that we demonstrate is similar to that from multicenter interventional trials from the same time period and also with different ICD-9 coding algorithms (Angus). In a recent meta-analysis detailing the mortality in patients with severe sepsis, patients receiving usual care in randomized controlled trials had a 28-day mortality of 33.2% that compares well with the overall mortality of 33.36% that we report [21]. Using the Angus algorithm (infection and acute organ dysfunction), Stevenson et al reported 31% mortality in 2009 that compares well with the 31.9% that we report in 2009 [21]. When restricting our cohort of patients with severe sepsis to those with septic shock, we saw a mortality rate of 39.8%, which compares well with the 38.4% reported by Levy et al. [2]. A similar pattern was seen when restricting to patients with severe sepsis who did not have septic shock.

Our study has several limitations. First, although the ICD-9-CM code that we used for severe sepsis has been validated, the inability to verify the accuracy of coding remains a limitation. The lack of a gold standard validation could mean that the actual incidence of severe sepsis is under or overestimated. Despite identifying the presence of severe sepsis in the cohort, ICD-9-CM codes cannot differentiate between the timing of infection and organ dysfunction. Hence, causality of severe sepsis and mortality cannot be firmly established. It is possible that the patient may have sustained another insult that eventually led to severe sepsis and death. Second, in the comparison between mortality between randomized clinical trials and our observational study, randomized trials report a lower mortality. These differences are likely due to exclusion criteria in clinical trials combined with patient-specific factors such as younger age observed in clinical trials. Even though the mortality rates that we identify compare well other studies using the NIS and other randomized clinical trials, the absolute 30- or 60-day mortality rate may not be comparable. Third, the higher mortality rates that we observe in lower tertile hospitals may occur if the goals of care are less aggressive in lower tertile hospitals that exclude transfer to a larger volume center. Fourth, other factors such ICU bed availability in patients with rapid clinical compromise may influence outcome independent of hospital volume.

In summary, we demonstrated an association between mean annual severe sepsis hospital volume and inpatient mortality. To effectively manage resources for improved outcomes in lower-volume hospitals we advocate a checklist-based approach for immediate stabilization and then transfer in the management of patients with severe sepsis [22].

## References

[1] Mayr FB, Yende S, Angus DC (2013) Epidemiology of severe sepsis. Virulence 5. 10.4161/viru.27372 PMC3916382 24335434

[2] Levy MM, Dellinger RP, Townsend SR, Linde-Zwirble WT, Marshall JC, et al (2010) The Surviving Sepsis Campaign: results of an international guideline-based performance improvement program targeting severe sepsis. Crit Care Med 38: 367–374. 20035219 10.1097/CCM.0b013e3181cb0cdc

[3] Chen YC, Chang SC, Pu C, Tang GJ (2013) The impact of nationwide education program on clinical practice in sepsis care and mortality of severe sepsis: a population-based study in Taiwan. PLoS One 8: e77414. 24124616 10.1371/journal.pone.0077414 PMC3790748

[4] Ferrer R, Artigas A, Levy MM, Blanco J, Gonzalez-Diaz G, et al (2008) Improvement in process of care and outcome after a multicenter severe sepsis educational program in Spain. JAMA 299: 2294–2303. 18492971 10.1001/jama.299.19.2294

[5] Birkmeyer JD, Sun Y, Wong SL, Stukel TA (2007) Hospital volume and late survival after cancer surgery. Ann Surg 245: 777–783. 17457171 10.1097/01.sla.0000252402.33814.dd PMC1877074

[6] Birkmeyer NJ, Dimick JB, Share D, Hawasli A, English WJ, et al (2010) Hospital complication rates with bariatric surgery in Michigan. JAMA 304: 435–442. 20664044 10.1001/jama.2010.1034

[7] Carey JS, Robertson JM, Misbach GA, Fisher AL (2003) Relationship of hospital volume to outcome in cardiac surgery programs in California. Am Surg 69: 63–68. 12575784

[8] Ross JS, Normand SL, Wang Y, Ko DT, Chen J, et al (2010) Hospital volume and 30-day mortality for three common medical conditions. N Engl J Med 362: 1110–1118. 20335587 10.1056/NEJMsa0907130 PMC2880468

[9] Shahin J, Harrison DA, Rowan KM (2012) Relation between volume and outcome for patients with severe sepsis in United Kingdom: retrospective cohort study. BMJ 344: e3394. 22645208 10.1136/bmj.e3394 PMC3362472

[10] Reinikainen M, Karlsson S, Varpula T, Parviainen I, Ruokonen E, et al (2010) Are small hospitals with small intensive care units able to treat patients with severe sepsis? Intensive Care Med 36: 673–679. 20143222 10.1007/s00134-009-1688-9

[11] Walkey AJ, Wiener RS (2014) Hospital Case Volume and Outcomes among Patients Hospitalized with Severe Sepsis. Am J Respir Crit Care Med 10.1164/rccm.201311-1967OC PMC3977716 24400669

[12] Kahn JM, Goss CH, Heagerty PJ, Kramer AA, O'Brien CR, et al (2006) Hospital volume and the outcomes of mechanical ventilation. N Engl J Med 355: 41–50. 16822995 10.1056/NEJMsa053993

[13] Walkey AJ, Wiener RS, Ghobrial JM, Curtis LH, Benjamin EJ (2011) Incident stroke and mortality associated with new-onset atrial fibrillation in patients hospitalized with severe sepsis. JAMA 306: 2248–2254. 22081378 10.1001/jama.2011.1615 PMC3408087

[14] Iwashyna TJ, Odden A, Rohde J, Bonham C, Kuhn L, et al (2012) Identifying Patients With Severe Sepsis Using Administrative Claims: Patient-Level Validation of the Angus Implementation of the International Consensus Conference Definition of Severe Sepsis. Med Care 10.1097/MLR.0b013e318268ac86 PMC3568444 23001437

[15] Quan H, Li B, Couris CM, Fushimi K, Graham P, et al (2011) Updating and validating the Charlson comorbidity index and score for risk adjustment in hospital discharge abstracts using data from 6 countries. Am J Epidemiol 173: 676–682. 21330339 10.1093/aje/kwq433

[16] Checkley W, Martin GS, Brown SM, Chang SY, Dabbagh O, et al (2014) Structure, process, and annual ICU mortality across 69 centers: United States Critical Illness and Injury Trials Group Critical Illness Outcomes Study. Crit Care Med 42: 344–356. 24145833 10.1097/CCM.0b013e3182a275d7 PMC4035482

[17] Hendryx MS, Fieselmann JF, Bock MJ, Wakefield DS, Helms CM, et al (1998) Outreach education to improve quality of rural ICU care. Results of a randomized trial. Am J Respir Crit Care Med 158: 418–423. 9700115 10.1164/ajrccm.158.2.9608068

[18] Kumar G, Kumar N, Taneja A, Kaleekal T, Tarima S, et al (2011) Nationwide trends of severe sepsis in the 21st century (2000–2007). Chest 140: 1223–1231. 21852297 10.1378/chest.11-0352

[19] Powell ES, Khare RK, Courtney DM, Feinglass J (2010) Volume of emergency department admissions for sepsis is related to inpatient mortality: results of a nationwide cross-sectional analysis. Crit Care Med 38: 2161–2168. 20802323 10.1097/CCM.0b013e3181f3e09c

[20] Djogovic D, Green R, Keyes R, Gray S, Stenstrom R, et al (2012) Canadian Association of Emergency Physicians sepsis treatment checklist: optimizing sepsis care in Canadian emergency departments. CJEM 14: 36–39. 22417956 10.2310/8000.2011.110610

[21] Stevenson EK, Rubenstein AR, Radin GT, Wiener RS, Walkey AJ (2014) Two decades of mortality trends among patients with severe sepsis: a comparative meta-analysis*. Crit Care Med 42: 625–631. 24201173 10.1097/CCM.0000000000000026 PMC4313930

[22] Djogovic D, Green R, Keyes R, Gray S, Stenstrom R, et al (2012) Canadian Association of Emergency Physicians sepsis treatment checklist: optimizing sepsis care in Canadian emergency departments. CJEM 14: 36–39. 22417956 10.2310/8000.2011.110610

